# The Nude at the Seaside

**Published:** 1891-08-08

**Authors:** 


### Passing Topics.

### THE NUDE AT THE SEASIDE.

Under this heading an American correspondent comments upon "the barbarous state of nakedness" in which children are permitted to display themselves at the seaside, and he adds that he cannot conceal his amazement that in so civilized a country as England "it should be taken as a matter of course for boys to run naked amid a promiscuous crowd of adults and young girls."

No doubt by the sea there is a tendency on the part of both sexes towards a relaxation of rules, strictly enforced elsewhere, which cannot be logically defended, but seems to be more or less taken for granted. Yet few men will deny that it is time this protest was made. Many people, doubtless, have uttered it to themselves and felt inclined to inflict summary chastisement upon the young rascals who offend with such brazen nonchalance, and if they have not taken the trouble to write to the papers, it is because they are a little sceptical as to the achievement by that means of any practical result. Nevertheless, something ought to be done to check a great and growing scandal, and if our American cousin's letter should serve to awaken the local authorities to their duty in this matter, every respectable frequenter of our watering places will rejoice. There need be no elaborate preparations. A short enactment conspicuously displayed and a couple of policemen to enforce its observance would ensure decency at once, and so far the end would be attained.

But is this enough? It is not only at the seaside, although there most flagrantly, that the rules of propriety are cast to the winds, and we are disposed to agree with the remark of the *Globe* that the male youth of the nation have much to learn on the score of "instinctive decency." It is to the cultivation of that quality rather than to police interference that we must look for really satisfactory results.

The question germane to this subject is whether the customary manner of dealing with our boys is not calculated to stifle, and ultimately to destroy that sense of propriety which, as our American friend rightly intimates, is essential to civilization? We must appeal to the mothers of England. It is for them to cultivate in their boys a regard for true—not prudish—modesty, and to respect and foster the diffidence which now too often exposes its possessor to ridicule and even censure. From the earliest years modesty in the male encounters unfavourable conditions. It is, we suppose, unavoidable that a middle-class boy must be handed over to the keeping of a not too refined nurse-maid, and that when he gets to be old enough to display feelings on the subject, they should be adjudged inconvenient, but it is surely unnecessary the lesson should be so carefully enforced that in time he comes to regard license as a prerogative of his kind. Women are a little too apt to claim modesty as an attribute of their own sex, and to be impatient of any pretension to it in the other. The average boy's mind becomes impressed with the notion that propriety is a poor, weak, effeminate virtue only to be practised by his sisters. We believe with our contemporary that there is such a thing as instinctive modesty, and it is because we believe in it that we suggest it should be given fair play.

Agricola.

